# The iron will of the research community: advances in iron nutrition and interactions in lockdown times

**DOI:** 10.1093/jxb/erab069

**Published:** 2021-03-16

**Authors:** Janneke Balk, Nicolaus von Wirén, Sebastien Thomine

**Affiliations:** 1 John Innes Centre, Colney Lane, Norwich NR4 7UH, UK; 2 Leibniz-Institute of Plant Genetics and Crop Plant Research (IPK), Gatersleben, Germany; 3 Université Paris-Saclay, CEA, CNRS, Institute for Integrative Biology of the Cell (I2BC), 91198, Gif-sur-Yvette, France

**Keywords:** Biofortification, iron homeostasis, micro-algae, natural variation, regulatory network, plant-microbe interactions

**Iron is an essential nutrient for most forms of life, and this is particularly the case for photosynthetic organisms such as plants and algae. Iron cofactors including haem and FeS clusters play key roles in photosynthetic electron transfer, as well as in electron transfer in the mitochondria to make ATP. The iron is, along with other mineral nutrients, obtained from the soil, and distributed where required. This is in contrast to mammals, like ourselves, who obtain iron from their food, including vegetables.**

The research field on iron nutrition in plants is very active, and has been for many decades while new discoveries are being made. An important forum for sharing these discoveries is the ISINIP meeting (International Symposium on Iron Nutrition and Interactions in Plants), which is held every 2 years. Unfortunately, due to the COVID-19 pandemic, the meeting planned for 1–5 July 2020 in Reims, France was postponed. The process of bringing out a special issue was already started, and is now complete, which is at least some comfort for those like ourselves who really missed coming together to discuss our favourite topics. This special issue contains 11 reviews and five research papers, which we hope give a good overview of the latest progress in the field.

In this Editorial, we highlight three themes, which reflect some of the most active or promising areas of research: (i) the regulation of iron homeostasis; (ii) plant–microbe interactions; and (iii) natural variation of iron uptake in different organisms in response to their environment. Outside these themes, the special issue also contains a review on the use of iron in mitochondrial FeS proteins (Przybyla-Toscano *et al.*, 2021) and three papers addressing different aspects of iron biofortification of food crops (Bashir *et al.*, 2021; Kawakami and Bhullar, 2021; Ram *et al*., 2021), which is a major application of the research field.

## The regulation of iron homeostasis

The amount of iron in the cell, and thus how much is taken up, incorporated into proteins, or stored, is tightly regulated. Not only a lack of iron but also an excess is detrimental, as is the case for most essential metals. The unique property of iron to oscillate between the ferrous (II) and ferric (III) state to accept or donate an electron also makes it a dangerous agent. When a plant is iron deficient, genes involved in iron acquisition by the roots are strongly induced; and when there is sufficient iron, storage mechanisms are activated. This is of course a simplified view, especially in a multicellular organism. It is perhaps no surprise that iron regulation is complex, and many transcription factors involved in iron homeostasis have been identified over the years. The main actors belong to the basic helix–loop–helix (bHLH) family of transcription factors, a gene family that has been exploited by plants to the full to regulate different processes, not just iron. Interestingly, in the last 2–3 years, a coherent framework is emerging that is central to orchestrating the transcriptional iron deficiency response in both dicot and monocot plants, as detailed in two of the reviews published in this issue (Gao and Dubos, 2021; Riaz and Guerinot, 2021). The bHLH transcription factors not only regulate each other’s transcriptional activity but also integrate other signals. In this issue, Von der Mark *et al.* (2021) show how reactive oxygen species, which accumulate under iron deficiency and in response to other stresses, repress iron uptake through a regulatory loop involving the transcription factor FIT and catalase. Post-translational regulation also plays a role in maintaining iron homeostasis. In particular, protein ubiquitination mediates the degradation of the core bHLH transcription factors and controls the recycling and degradation of the main iron transporter IRT1 (Dubeaux *et al.*, 2018; Spielmann and Vert, 2021). Mechanisms for how the iron status of the cell is sensed are also emerging. One of them involves the plant-specific BTS/L ubiquitin ligases that can bind iron (Rodríguez-Celma *et al.*, 2019; Spielmann and Vert, 2021). This proposed sensing mechanism is distinct from those in yeast and mammalian cells. On the other hand, the IMA peptides that accumulate at extremely high levels under Fe deficiency may mediate long-distance signalling of Fe deficiency (Grillet *et al.*, 2018). A research paper in this issue analyses the roles of IMA peptides in rice (Kobayashi *et al.*, 2021).

## Iron in plant–microbe interactions

Plants do not live in a sterile (laboratory) environment, but constantly interact with ‘good’ and ‘bad’ microbes. For a long time, it has been clear that iron plays an important role in plant–microbe interactions, but these are complex biological systems to study. Partly as a consequence of the availability of genome sequences and omics data, as well as advanced molecular tools, this topic in iron research has expanded and is covered by a review (Liu *et al.*, 2021). Iron can be a real bargaining chip, with microbes needing it for infection and growth, whereas plants will try to actively withhold it. In this issue, Trapet *et al.*, (2021) show that iron deficiency in a plant limits infection by pathogens with different lifestyles, ranging from necrotrophic to biotrophic. Using Arabidopsis mutants in iron uptake, they show that resistance to these pathogens is not simply caused by making iron scarce, but is a plant-mediated defence response that requires ethylene and salicylic acid signalling. On the other hand, iron, due to its ability to generate reactive oxygen species, may also be used to kill the invader or execute cell death to prevent pathogen progression.

Regulated cell death is often part of a defence response in plants, and, in some cases, iron may be involved in triggering cell death. Iron-dependent cell death, named ferroptosis, was recently described in mammalian cells and the same features have been observed in plants in the context of plant–pathogen interactions, as reviewed in this issue (Distefano *et al.*, 2021).

Whereas plants have developed strategies to withdraw iron from pathogens, they must provide it to those microbes they host in symbiotic or endophytic associations. Symbiotic rhizobia in root nodules of legumes constitute a major sink for iron as a cofactor of nitrogenase and many other enzymes required for fixation of atmospheric nitrogen. Although not covered in this issue, our knowledge on transporters involved in providing iron to intracellular bacteria has substantially increased over the past couple of years. Functional studies in several legume species have shown that MATE proteins homologous to FRD3 in Arabidopsis facilitate iron transport from the nodule xylem into the nodule (Wang *et al.*, 2017; Kryvoruchko *et al.*, 2018). An NRAMP homologue then transports iron into the infected cell, and VIT-like transporters export the iron into the peribacteroid space (Brear *et al.*, 2020; Liu *et al.*, 2020; Walton *et al.*, 2020), from where the nitrogen-fixing bacteria can take it up with as yet unknown transporters.

## Natural variation of iron uptake in different environments

The solubility of iron is strongly influenced by both redox potential and pH, and this in turn greatly affects iron uptake. Organisms adapt by switching between different uptake mechanisms, mainly free Fe^2+^ or Fe(III)-chelates, or high-affinity and low-affinity transport systems. This is well studied in bacteria, but how plants adapt their iron uptake mechanisms to different soil conditions is only recently receiving more attention. Aside from pH, the abundance of other metals and nutrients in the environment not only affects iron uptake but may interact with iron at multiple levels (Hanikenne *et al.*, 2021). Genetic variation in iron uptake mechanisms may be used for breeding more resilient crops. Rice grown in wet paddies, in which iron is present as the highly soluble Fe^2+^ species, often suffers from iron toxicity. In this issue, a paper by Frei and colleagues reports the identification of quantittive trait loci (QTLs) for iron tolerance in an interspecies cross between cultivated rice and a related species adapted to high-iron soil. The study identifies genes putatively involved in iron tolerance that could be bred into crop varieties (Wairich *et al.*, 2021).

Natural populations of the same species growing in different soils have also adapted over evolutionary time scales. Studies of intraspecific variation are beginning to show how allelic variation in the plant genome tunes some iron responses, such as root ferroxidase activity or the rate of coumarin secretion, and thus drives adaptation to iron-deficient environments, for example on calcareous soils (Miller and Busch, 2021). On the other hand, very little is known about evolutionary differences in the mechanisms of iron acquisition and homeostasis in the green lineage. In this respect, a lot is to be learned from iron homeostasis mechanisms in algae, which have to deal with constantly changing environmental conditions and extreme fluctuations in the abundance of iron (Gao *et al.*, 2021).

## Future perspectives

The field of iron nutrition and interactions in plants has made tremendous progress and continues opening new areas of investigation. Nevertheless, there is a need for a better understanding of the biochemical aspects of iron homeostasis, such as protein interactions in iron uptake (Martín-Barranco *et al.*, 2020) and iron signalling, and how iron is trafficked and incorporated in iron-dependent proteins or photosystems. A community resource to catalogue all iron-binding proteins and their function has recently been set up (Przybyla-Toscano *et al.*, 2021).

Although the role of rhizosphere microorganisms in iron acquisition has received particular attention, there is still the need to amplify research in this direction, for example the contribution of mycorrhizae. Beneficial microbes have been shown to strengthen plant defence using components that are shared with the iron homeostasis system (Stringlis *et al.*, 2018). How the iron nutritional status of the plant influences rhizosphere communities through the secretion of coumarins and other organic compounds remains largely unexplored, but bears potential to inform agroecological field practice (Tsai and Schmidt, 2017).

Another future line of exploration is the diversity in iron acquisition and homeostasis mechanisms in the green lineage. Most of our knowledge comes from the study of the two higher plant species Arabidopsis and rice, and the algal species *Chlamydomonas*. Looking at the amount of variation among related species and intraspecific variation, as illustrated in this issue, one may wonder if Arabidopsis is representative of all dicotyledons, and if rice, besides its interest as a major crop, is representative of all gramineous species. Another almost virgin field of investigations is the diversity of iron homeostasis mechanisms in algae. The scarcity of iron in the ocean and the phylogenetic and ecological diversity of algae are likely to be associated with a wealth of unsuspected mechanisms, as illustrated for diatoms in this issue. Their elucidation will be crucial for understanding iron geochemistry and iron cycling in the oceans and how it controls productivity and CO_2_ fixation. It may also provide new concepts to improve iron nutrition in land plants.

## References

[CIT0001] Bashir K, Ahmad Z, Kobayashi T, Seki M, Nishizawa NK. 2021. Roles of subcellular metal homeostasis in crop improvement. Journal of Experimental Botany 72, 2083–2098. 10.1093/jxb/erab018 33502492

[CIT0002] Brear EM, Bedon F, Gavrin A, Kryvoruchko IS, Torres-Jerez I, Udvardi MK, Day DA, Smith PMC. 2020. GmVTL1a is an iron transporter on the symbiosome membrane of soybean with an important role in nitrogen fixation. New Phytologist 228, 667–681. 10.1111/nph.16734 32533710

[CIT0003] Distefano AM, Lopez GA, Setzes N, Marchetti F, Cainzo M, Cascallares M, Zabaleta E, Pagnussat GC. 2021. Ferroptosis in plants: triggers, proposed mechanisms, and the role of iron in modulating cell death. Journal of Experimental Botany 72, 2125–2135. 10.1093/jxb/eraa425 32918080

[CIT0004] Dubeaux G, Neveu J, Zelazny E, Vert G. 2018. Metal sensing by the IRT1 transporter-receptor orchestrates its own degradation and plant metal nutrition. Molecular Cell 69, 953–964.e5. 29547723 10.1016/j.molcel.2018.02.009

[CIT0005] Gao X, Bowler C, Kazamia E. 2021. Iron metabolism strategies in diatoms. Journal of Experimental Botany 72, 2165–2180. 10.1093/jxb/eraa575 PMC7966952 33693565

[CIT0006] Gao F, Dubos C. 2021. Transcriptional integration of plant responses to iron availability. Journal of Experimental Botany 72, 2056–2070. 10.1093/jxb/eraa556 33246334

[CIT0007] Grillet L, Lan P, Li W, Mokkapati G, Schmidt W. 2018. IRON MAN is a ubiquitous family of peptides that control iron transport in plants. Nature Plants 4, 953–963. 30323182 10.1038/s41477-018-0266-y

[CIT0008] Hanikenne M, Esteves SM, Fanara S, Rouached H. 2021. Coordinated homeostasis of essential mineral nutrients: a focus on iron. Journal of Experimental Botany 72, 2136–2153. 10.1093/jxb/eraa483 33175167

[CIT0009] Kawakami Y, Bhullar NK. 2021. Delineating the future of iron biofortification studies in rice: challenges and future perspectives. Journal of Experimental Botany 72, 2099–2113. 10.1093/jxb/eraa446 32974681

[CIT0010] Kobayashi T, Nagano AJ, Nishizawa NK. 2021. Iron deficiency-inducible peptide-coding genes *OsIMA1* and *OsIMA2* positively regulate a major pathway of iron uptake and translocation in rice. Journal of Experimental Botany 72, 2196–2211. 10.1093/jxb/eraa546 33206982

[CIT0011] Kryvoruchko IS, Routray P, Sinharoy S, et al. 2018. An iron-activated citrate transporter, MtMATE67, is required for symbiotic nitrogen fixation. Plant Physiology 176, 2315–2329. 29284744 10.1104/pp.17.01538 PMC5841734

[CIT0012] Liu Y, Kong D, Wu H-L, Ling H-Q. 2021. Iron in plant–pathogen interactions. Journal of Experimental Botany 72, 2114–2124. 10.1093/jxb/eraa516 33161430

[CIT0013] Liu S, Liao LL, Nie MM, Peng WT, Zhang MS, Lei JN, Zhong YJ, Liao H, Chen ZC. 2020. A VIT-like transporter facilitates iron transport into nodule symbiosomes for nitrogen fixation in soybean. New Phytologist 226, 1413–1428. 10.1111/nph.16506 32119117

[CIT0014] Martín-Barranco A, Spielmann J, Dubeaux G, Vert G, Zelazny E. 2020. Dynamic control of the high-affinity iron uptake complex in root epidermal cells. Plant Physiology 184, 1236–1250. 32873629 10.1104/pp.20.00234 PMC7608170

[CIT0015] Miller CN, Busch W. 2021. Using natural variation to understand plant responses to iron availability. Journal of Experimental Botany 72, 2154–2164. 10.1093/jxb/erab012 PMC7966951 33458759

[CIT0016] Przybyla-Toscano J, Boussardon C, Law S, Rouhier N, Keech O. 2021. Gene atlas of iron-containing proteins in *Arabidopsis thaliana*. The Plant Journal. doi: 10.1111/tpj.15154. 33423341

[CIT0017] Przybyla-Toscano J, Christ L, Keech O, Rouhier N. 2021. Iron–sulfur proteins in plant mitochondria: roles and maturation. Journal of Experimental Botany 72, 2014–2044. 10.1093/jxb/eraa578 33301571

[CIT0018] Ram H, Singh A, Katoch M, et al. 2021. Dissecting the nutrient partitioning mechanism in rice grain using spatially resolved gene expression profiling. Journal of Experimental Botany 72, 2212–2230. 10.1093/jxb/eraa536 33197257

[CIT0019] Riaz N, Guerinot ML. 2021. All together now: regulation of the iron deficiency response. Journal of Experimental Botany 72, 2045–2055. 10.1093/jxb/erab003 PMC7966950 33449088

[CIT0020] Rodríguez-Celma J, Connorton JM, Kruse I, Green RT, Franceschetti M, Chen Y-T, Cui Y, Ling H-Q, Yeh K-C, Balk J. 2019. Arabidopsis BRUTUS-LIKE E3 ligases negatively regulate iron uptake by targeting transcription factor FIT for recycling. Proceedings of the National Academy of Sciences, USA 116, 17584–17591. 10.1073/pnas.1907971116 PMC6717287 31413196

[CIT0021] Spielmann J, Vert G. 2021. The many facets of protein ubiquitination and degradation in plant root iron deficiency responses. Journal of Experimental Botany 72, 2071–2082. 10.1093/jxb/eraa441 32945865

[CIT0022] Stringlis IA, Yu K, Feussner K, de Jonge R, Van Bentum S, Van Verk MC, Berendsen RL, Bakker PAHM, Feussner I, Pieterse CMJ. 2018. MYB72-dependent coumarin exudation shapes root microbiome assembly to promote plant health. Proceedings of the National Academy of Sciences, USA 115, E5213–E5222. 10.1073/pnas.1722335115 PMC5984513 29686086

[CIT0023] Trapet PL, Verbon EH, Bosma RR, Voordendag K, Van Pelt JA, Pieterse CMJ. 2021. Mechanisms underlying iron deficiency-induced resistance against pathogens with different lifestyles. Journal of Experimental Botany 72, 2231–2241. 10.1093/jxb/eraa535 33188427

[CIT0024] Tsai HH, Schmidt W. 2017. Mobilization of iron by plant-borne coumarins. Trends in Plant Science 22, 538–548. 28385337 10.1016/j.tplants.2017.03.008

[CIT0025] von der Mark C, Ivanov R, Eutebach M, Maurino VG, Bauer P, Brumbarova T. 2021. Reactive oxygen species coordinate the transcriptional responses to iron availability in Arabidopsis. Journal of Experimental Botany 72, 2181–2195. 10.1093/jxb/eraa522 PMC7966954 33159788

[CIT0026] Wairich A, de Oliveira BHN, Wu LB, Murugaiyan V, Margis MP, Fett JP, Ricachenevsky F, Frei M. 2021. Chromosomal introgressions from *Oryza meridionalis* into domesticated rice *Oryza sativa* result in iron tolerance. Journal of Experimental Botany 72, 2242–2259. 10.1093/jxb/eraa461 33035327

[CIT0027] Walton JH, Kontra-Kováts G, Green RT, Domonkos Á, Horváth B, Brear EM, Franceschetti M, Kaló P, Balk J. 2020. The *Medicago truncatula* Vacuolar iron Transporter-Like proteins VTL4 and VTL8 deliver iron to symbiotic bacteria at different stages of the infection process. New Phytologist 228, 651–666. 10.1111/nph.16735 PMC7540006 32521047

[CIT0028] Wang J, Hou Q, Li P, Yang L, Sun X, Benedito VA, Wen J, Chen B, Mysore KS, Zhao J. 2017. Diverse functions of multidrug and toxin extrusion (MATE) transporters in citric acid efflux and metal homeostasis in *Medicago truncatula*. The Plant Journal 90, 79–95. 28052433 10.1111/tpj.13471

